# Identifying Positive and Negative Factors That Affect the Promotion of Clinical Faculty at the Wayne State University School of Medicine: Does Gender Matter?

**DOI:** 10.7759/cureus.29954

**Published:** 2022-10-05

**Authors:** Anne Messman, R. B Stansfield, Yang Liu, John Collins, Marissa Matthews, Robert Ehrman

**Affiliations:** 1 Department of Emergency Medicine, Wayne State University, Detroit, USA; 2 Department of Graduate Medical Education, Wayne State University School of Medicine, Detroit, USA; 3 Department of Emergency Medicine, Sinai-Grace Hospital, Detroit, USA; 4 Department of Emergency Medicine, Wayne State University School of Medicine, Detroit, USA

**Keywords:** faculty development, gender, promotion, research, medical education

## Abstract

Introduction: Despite well-documented gender disparity in academic medicine, there are many women who achieve success, including successful promotion to associate and full professor status. This study sought to determine whether there was a gender difference in the perception of positive and negative factors affecting the process of promotion to associate or full professor at the Wayne State University School of Medicine (WSUSOM).

Methods: All clinically active associate and full professors who achieved their most recent promotion at the WSUSOM were sent a link to a survey that obtained demographic information as well as the opinions of the respondents regarding what positive and negative factors impacted their most recent promotion.

Results: Of the 73 respondents (24%), 58 (19%) were included in our final analysis. Two obstacles (“Lack of interest and encouragement from institutional or departmental leaders” and “Lack of tangible commitment from institutional or departmental leadership [e.g., protected time]”) were ranked in the top three ranks by a substantially greater percentage of females than males. Gender-specific networking was seen as significantly more valuable to female faculty members whereas having a stay-at-home partner was seen as significantly more valuable to male faculty members.

Conclusion: At the WSUSOM, providing more gender-specific networking for women, increasing interest and encouragement from institutional and departmental leaders, and providing a tangible commitment to female faculty from these leaders may help more women to achieve promotion to associate or full professor.

## Introduction

In academic medicine, the female gender has been associated with lower salaries, fewer scholarly opportunities, and a lower likelihood of achieving full professor status, even after adjusting for years in practice [1-7]. The female gender has also been found to be negatively associated with having a major leadership role [3,7-9]. Although these appear to be slowly improving, there are well-documented gender disparities that start upon entry into the job market and persist regardless of age, years since residency completion, or research productivity [2]. Additionally, progress made towards gender parity over recent years may be adversely affected by the coronavirus disease 2019 (COVID-19) pandemic due to unequal division of household labor and family care during the pandemic, particularly if the physician is a mother [10,11]. Despite these significant impediments to career advancement at nearly every level, there are women who attain associate professor positions and beyond. Identification of factors that helped or hindered these women in the promotion process will give departments and institutions direction, so they know where to invest more resources and where there is room for improvement.

A 2019 study by Siddle et al. used an exploratory survey to identify positive and negative influences on female first authorship in the journal Academic Emergency Medicine, a leading journal for scientific emergency medicine research. Their sample consisted of all published first authors in the journal Academic Emergency Medicine over a five-year period. The authors found that women valued mentoring, departmental policies, and empowerment in discussions as “very important” when compared to males [12]. Additionally, surveys from the American National Academy of Sciences assessed for gender differences in barriers to workplace advancement and success. They found that lack of mentorship, limited understanding of promotional guidelines, and lack of protected academic time were found to affect women differently [13].

These differences were once again illustrated in a publication by Jasgsi et al., which showed that women had shorter one-on-one meeting time with mentors when compared to men and perceived less collegiality in the mentorship relationship. While female respondents were provided with similar space and equipment as their male counterparts, they were met with less access to human resources like secretaries, grant administrators, and statisticians [14]. All of these, taken together, may contribute to the findings of Speck et al. who found that women were at much greater risk than men on the clinician-educator track of leaving their job as junior faculty prior to their first promotion, significantly slowing their professional development [15].

With these prior studies in mind, we sought to determine whether there was a gender difference in perception of positive and negative factors affecting the process of promotion to associate or full professor at the Wayne State University School of Medicine (WSUSOM).

## Materials and methods

Setting and participants

This study took place at the WSUSOM in Detroit, Michigan from April to May 2021. All full-time associates and full professors who also practice clinically were invited to participate in the study (n=301). Participants were excluded if they did not meet the aforementioned inclusion criteria or if their most recent promotion to either associate or full professor took place at an institution other than the WSUSOM.

Survey

The survey was adapted with permission from a prior study examining whether there were gender differences in positive and negative influences in the first authorship in a major academic emergency medicine journal [12]. Our adapted survey was content validated by two experts in survey design. It was subsequently piloted by two participants who were not part of the final study cohort. The final version of the survey can be found in the appendices. The survey was distributed via link to a Google Form to potential participants via their WSUSOM email address. Reminder emails were sent one and two weeks after the initial email. The study was closed three weeks after the initial email. Participation in the survey was anonymous, voluntary, and uncompensated. 

Data analysis

Demographics

Respondents were excluded if they did not consent to have their data used for research purposes, if their most recent academic promotion had not been at the WSUSOM, or if they were not clinical-track faculty at the time of the survey. Respondents were asked to indicate with which gender they identify, and the sample was compared by gender, age, marital status, parenthood, academic rank, and reported number of years to achieve their most recent promotion.

Obstacles

Obstacle rankings were coded from 1 (“1st [largest obstacle]”) to 5 (“Less important >4”). Median rank of each obstacle was calculated and compared between genders using the Wilcoxon Rank-Sum test. Additionally, the percentage of faculty who ranked each obstacle in the first, second, or third rank was computed for each gender. Only obstacles that were rated by each subject were included in the calculations.

Contributions

Contribution ratings were coded from 0 (“No contribution at all”) to 4 (“Very important contribution”). Median ratings were computed for each gender and compared using the Wilcoxon Rank-Sum test. Frequency data are presented as percentages and continuous data as medians with range and inter-quartile range (IQR). Statistical analyses were performed using SAS v9.4 (SAS Institute, Cary, NC), and R v3.6.3, with the significance level set at 0.05.

Institutional review board approval

The study was approved by the WSUSOM Institutional Review Board (IRB-20-10-2807).

## Results

Demographics

Of the 73 (24%) respondents, two were removed for non-consent, seven were removed because their most recent promotion was not at the WSUSOM, and six were removed because they were not clinical-track faculty at the WSUSOM at the time of the survey. This resulted in 58 (19%) respondents: 31 identified as male (53%) and 27 as female (47%). 

The modal age bracket was 50-59 years for the whole sample (33%) although the females were slightly older (73% of females were aged ≥ 50 compared to 61% of males). Overall, most participants were married (88%) while 15% of women reported never having been married compared to only 3% of men. A total of 62% of respondents had children or dependents living at home at least part-time, while the percentage was slightly greater for men (68%) compared to women (56%). A total of 14 participants held advanced degrees, with seven PhDs (4% of females and 20% of males) and seven master’s degrees (15% of females, 10% of males); data were missing for one subject in this response. Fellowship training was completed by 42 people overall, with a slightly greater proportion of males (57% versus 42%). Twenty-seven subjects (52% of females and 48% of males) self-identified as belonging to at least one of the following minority groups: Lesbian, Gay, Bisexual, Transgender, and Queer/Questioning (LGBTQ), international medical graduate, religious minority, or non-Caucasian race or ethnicity.

A majority of respondents were full professors (57% overall, 55% of males, and 59% of females) and the remainder were at the associate professor level. Overall, 26% of subjects had been faculty at the WSUSOM for less than 10 years, with a greater proportion of males in this category compared to females (32% versus 19%). The overall median reported time to achieve the most recent promotion was 7 (range 1-25, inter-quartile range [IQR] 5) years; for females, the median was 7.5 (range 4-25, IQR 7) years and for males, the median was 6.5 (range 1-21, IQR 4) years. Data on years to the most recent promotion were missing for two subjects.

Obstacles

Table 1 shows the by-gender comparison median rank, and percentage with ranking in the top three, for each obstacle. Two obstacles (“Lack of interest and encouragement from institutional or departmental leaders” and “Lack of tangible commitment from institutional or departmental leadership [e.g., protected time]”) were ranked in the top three ranks by a substantially greater percentage of females than males. The remaining obstacles were more similarly ranked in the top three by both genders.

**Table 1:** Comparison by gender of obstacle rankings from 1 (“1st [most important]”) through 5 (“Less important [>4]”). For each obstacle, the first column for each gender represents the percent of respondents ranking each between 1 and 3; the second column under each gender reports the number of respondents who ranked that obstacle followed by the median and interquartile range (N/median [IQR]). * indicates a statistically significant p-value P-values are for comparison of medians using the Wilcoxon Rank-Sum test.

Obstacle	Male		Female		Wilcoxon Rank Sum Test (p-value)
Lack of interest and encouragement from institutional or departmental leaders	38.7%	26/4(3)	74.1%	24/2(2)	0.004*
Lack of tangible commitment from institutional or departmental leadership (e.g. protected time)	45.2%	23/2(2)	66.7%	21/2(2)	0.15
Competing administrative responsibilities	48.4%	27/3(2)	48.1%	18/3(2)	0.45
Lack of mentorship	41.9%	26/3.5(3)	40.7%	19/3(3)	0.35
Lack of resources to support my work, such as statistical or personnel support	48.4%	27/3(3)	40.7%	18/3(2)	0.38
Competing domestic responsibilities	29.0%	24/4.5(2)	25.9%	17/4(4)	0.47
Lack of my own interest or motivation	19.4%	22/5(2)	14.8%	12/4.5(2)	0.40
Lack of interest and encouragement from those in my personal life	12.9%	21/5(1)	14.8%	11/5(2)	0.31

Contributions

Table 2 shows the median rating of each contribution for males and females and the Wilcoxon Rank-Sum test results for each contribution.

**Table 2:** Ratings of contributions to promotion with the median value reported first followed by inter-quartile range in parenthesis for male and female faculty. * indicates a statistically significant p-value P-values represent comparison of medians using the Wilcoxon Rank-Sum test.

Item	Male	Female	WRS test (p-value)
Gender-specific networking	0(0)	1(2)	0.01*
Mentoring from a person sought by you	3(1)	3(3)	0.49
My own determination	4(1)	4(0)	0.39
My ability to negotiate/be seen as equal	2(2)	2(2)	0.85
Formal research training	2(4)	2(3.5)	0.89
Formal fellowship training	2(3)	1.5(4)	0.70
Protection from clinical duties by funding	1(2)	0(2)	0.21
Mentoring from someone assigned to you	0(2)	0(1)	0.43
Direct reward for scholarship	0(0)	0(0)	0.20
Policies requiring scholarship for rank	2(3)	1(2)	0.11
Departmental resources	2(3)	1(2)	0.12
Protection from clinical duties by department	1(3)	0(1)	0.04*
Having a stay-at-home partner	2(3)	0(0)	0.003*

Both genders had similarly strong feelings that their own drive and determination were very important, as was guidance from self-sought mentors. Moderately important factors for both genders included being seen as equal with other departmental decision-makers and having formal research or fellowship training. Financial reward for scholarly productivity was rated as unimportant for both females and males. Males rated protection from clinical duties by their department as slightly more important than females while having a stay-at-home partner was at least moderately important for males but not important for females.

## Discussion

This study demonstrates a gendered difference in both positive and negative factors that affected the promotion process to associate and full professor at the WSUSOM. This is consistent with prior research in the field and serves to strengthen these prior findings.

The WSUSOM is representative of many schools of medicine in the United States wherein women are underrepresented at all levels of academic ranking. Per the WSUSOM’s dean’s office, women comprise 40% of assistant professors, 33% of associate professors, and just 23% of full professors at the WSUSOM. Understanding the experiences of the women who have successfully navigated promotion to associate and full professor will allow the WSUSOM to target specific initiatives to maximize positive experiences that were helpful in the promotion process and mitigate those perceived as obstacles to promotion. For example, “gender-specific networking” was cited by women as a significantly more important positive factor in their most recent promotion as compared to their male counterparts, a finding supported by other work in the field [12]. The WSUSOM should therefore focus attention to increase these networking opportunities for women. Given the current restrictions in place because of the COVID-19 pandemic, these networking opportunities are threatened and limited; the School of Medicine must take special care to ensure that these opportunities are still in place, even if they must be virtual.

Conversely, several obstacles were perceived to be significantly larger barriers to promotion by women as opposed to their male counterparts. “Lack of interest and encouragement from institutional or departmental leaders,” and “Lack of tangible commitment from institutional or departmental leadership (e.g., protected time)” are factors that can be mitigated by both the WSUSOM and individual departments within the school. This valuable feedback will be shared with department chairs at the WSUSOM to ensure that they are aware of the perception of these obstacles by their female faculty. Likewise, leadership at the WSUSOM needs to act to mitigate these negative effects on the promotion of women.

Although the focus of this study was to highlight ways to enhance the promotion of women in academic medicine, the study also yields data that can help in the promotion of men at the WSUSOM. This study did find two factors that were significantly helpful to men in their most recent promotion, namely “Protection from clinical duties by the department” and “Having a stay-at-home partner.” While it is clearly not in the purview of the WSUSOM to encourage male faculty to have a stay-at-home partner, department chairs can re-examine the amount of protected time that their male faculty members have and ensure, when reasonable, that a lack of protected time is not inhibiting the promotion of their male faculty members.

Limitations

Limitations of this study include a relatively low response rate and its single-site nature. It is important to note that while this study was performed at a single institution, it did reproduce themes that have been demonstrated in prior work in this field, suggesting that these themes are likely prevalent at other academic institutions beyond the WSUSOM. In addition, the creation of our survey instrument may be of use to other schools of medicine that may wish to repeat this study at their own institution to identify their own unique challenges. Future research should focus on multi-site validation of our findings and whether targeting positive and negative promotion factors, for example increasing the amount and availability of gender-specific networking, results in an increase in the promotion of female faculty. Qualitative research may also reveal additional positive and negative factors that affect the promotion process that was not listed in our survey.

## Conclusions

At the WSUSOM, providing more gender-specific networking for women, increasing interest and encouragement from institutional and departmental leaders, and providing a tangible commitment to female faculty from these leaders may help more women to achieve promotion to associate or full professor. These findings may help other academic institutions target interventions that may help in the promotion of female faculty members. In addition, institutions may find our survey instrument to be useful in order to repeat the study with their own faculty members. Our hope is that our findings and survey instrument will help to achieve gender parity in terms of promotion in academic medicine.

## Appendices

Did your most recent promotion take place at the Wayne State University School of Medicine?

Demographics

**Table 3:** Demographic survey

Demographics			
	Female	Male	Other
What is your gender?	☐	☐	☐
				If you had a primary mentor, what was that person’s gender?	☐	☐	☐
	☐	0-5 years	
How many years have you been faculty at Wayne State University School of Medicine? (If you were initially voluntary faculty then became non-voluntary faculty, please only include the years since you became non-voluntary faculty)	☐	6-10 years	
	☐	11-15 years	
	☐	16-20 years	
	☐	20+ years	
Since you became non-voluntary faculty at Wayne State University School of Medicine, how many years did it take to achieve your most recent promotion to associate/full professor. Please round to the nearest year. Promotion is “achieved” when the promotion was officially conferred to you (usually in August), not at the time that your application was submitted.	(Free text response)		
	☐	Associate Professor	
What is your current academic rank?	☐	Full Professor	
	☐	Other	
	☐	20-29 years	
	☐	30-39 years	
	☐	40-49 years	
What is your age range?	☐	50-59 years	
	☐	60-69 years	
	☐	70-79 years	
	☐	80 years or greater	
	☐	Single or Never Married	
	☐	Married	
What is your current marital status?	☐	Widowed	
	☐	Divorced or Separated	
	☐	None of the above	
	☐	No	
Do you have children or dependents?	☐	Yes, living in my home	
	☐	Yes, living with me part-time	
	☐	Yes, living predominantly outside of my home	
	☐	MD or DO	
Mark your degree(s)	☐	PhD	
	☐	Masters degree	
	☐	Other advanced degree (please specify)	

1.    Please rate how strongly each of the factors below contributed to you achieving status as an associate or full professor.

**Table 4:** Contributing factors to promotion

	Not at all		Somewhat		Very Much	N/A (question does not apply)
Formal research training (e.g., research fellowship, research training grant)	☐	☐	☐	☐	☐	☐
Formal fellowship training other than research	☐	☐	☐	☐	☐	☐
Mentoring from a person or persons sought out by you	☐	☐	☐	☐	☐	☐
Mentoring from a scholarly mentoring panel, assembled by someone other than yourself	☐	☐	☐	☐	☐	☐
Gender specific networking (e.g., women’s peer support)	☐	☐	☐	☐	☐	☐
Protection from clinical duties provided by grant funding	☐	☐	☐	☐	☐	☐
Protection from clinical duties provided by your department/division, independent of grant funding	☐	☐	☐	☐	☐	☐
Departmental or institutional resources such as technical, intellectual, and staff support that facilitate scholarship (e.g., help with statistics or regulatory processes)	☐	☐	☐	☐	☐	☐
Direct departmental or institutional fiscal reward for scholarship (e.g., payment per manuscript)	☐	☐	☐	☐	☐	☐
Departmental or institutional policies that require scholarship for rank advancement, salary increase or promotion and tenure	☐	☐	☐	☐	☐	☐
My own determination and drive	☐	☐	☐	☐	☐	☐
Having a stay at home significant other, spouse or partner to help with domestic responsibilities	☐	☐	☐	☐	☐	☐
My ability to negotiate or be seen as an equal in discussions with other decision makers of my department/division	☐	☐	☐	☐	☐	☐

Please rank FOUR of the following reasons as the OBSTACLES to attaining your most recent promotion to associate/full professor with "First" representing the largest obstacle. You can select “Less important (<4)” for all remaining items that are less important than the first four.

**Table 5:** Obstacles to promotion

	First	Second	Third	Fourth	Less important (<4)
Competing domestic responsibilities	☐	☐	☐	☐	☐
Competing administrative responsibilities	☐	☐	☐	☐	☐
Lack of my own interest or motivation	☐	☐	☐	☐	☐
Lack of mentorship	☐	☐	☐	☐	☐
Lack of interest and encouragement from institutional or departmental leaders	☐	☐	☐	☐	☐
Lack of interest and encouragement from those in my personal life	☐	☐	☐	☐	☐
Lack of tangible commitment from institutional or departmental leadership (e.g. protected time)	☐	☐	☐	☐	☐
Lack of resources to support my work, such as statistical or personnel support	☐	☐	☐	☐	☐
